# Response to “COVID-19: room for treating T cell exhaustion?”

**DOI:** 10.1186/s13054-020-03068-1

**Published:** 2020-06-15

**Authors:** Anne M. Drewry, Richard Hotchkiss, Erik Kulstad

**Affiliations:** 1 Department of Anesthesiology, Washington University School of Medicine, 660 S. Euclid Ave., Campus Box 8054, St. Louis, MO 63110 USA; 2 Anesthesiology, Medicine, Surgery, and Developmental Biology, Washington University School of Medicine, 660 S. Euclid Ave., Campus Box 8054, St. Louis, MO 63110 USA; 3 Department of Emergency Medicine, UT Southwestern Medical Center, 5323 Harry Hines Blvd., Dallas, TX 75390 USA

Dear Editor,

Riva et al. elegantly discuss the complex balance between immunosuppressive and immunostimulating factors in the treatment of COVID-19 and raise the point that therapeutic approaches to enhance immune function, particularly T cell functions, have not yet been attempted in this setting [1]. Recent data describing circulating SARS-CoV-2-specific CD8+ and CD4+ T cells in COVID-19 convalescent patients further support the potential utility of enhancing T cell activity [2].

One additional factor to consider is the influence of patient temperature on immune function. Several studies show that elevated temperature boosts multiple aspects of both humoral and cellular immunity, including antibody production, T cell activation, and macrophage function. Fever promotes T lymphocyte trafficking through heat shock protein 90 (Hsp90)-induced α4 integrin activation and signaling in T cells, promoting T cell adhesion and migration to enhance immune surveillance during infection [3]. A retrospective review of over 400 patients with sepsis suggests that moderate fever (38.3–39.4 °C) is protective [4]. Prospective data have shown that afebrile patients have higher 28-day mortality (37.5% vs 18.2%), increased acquisition of secondary infections (35.4% vs. 15.9%), and suppressed HLA-DR expression over time (a finding suggestive of monocyte dysfunction in sepsis) [5]. A pilot randomized controlled study of external warming of septic patients (ClinicalTrials.gov identifier: NCT02706275) has recently completed enrollment.

The importance of patient temperature, and the potential for benefits from warming in a variety of infectious conditions, including COVID-19, warrants continued study. A recently posted randomized controlled trial protocol is a further step towards this goal (https://www.medrxiv.org/content/10.1101/2020.04.03.20052001v1).

## Authors’ response

Giovanni Riva, Vincenzo Nasillo, Enrico Tagliafico, Tommaso Trenti, Mario Luppi

Dear Editor,

We read with interest the comment by Drewry and colleagues, pointing out the relevant effects of patient temperature on immune function. In particular, the authors remark the potential improvement of pathogen-specific T cell responses and innate immunity associated with 1–2° increase of body temperature (i.e., moderate fever), which appeared to be correlated with a better outcome in critically ill infected patients, suggesting that external “core warming” may represent a valuable non-pharmaceutical intervention to enhance anti-pathogen immunity in septic patients, including those with COVID-19.

In line with this observation, it has recently been highlighted that climatic environmental factors (such as low temperatures and dry air) directly influence clinico-epidemiological manifestations of respiratory virus infections (included coronaviruses), showing typical outbreaks in the cold season, while vanishing or just causing mild symptoms in the summer months. Indeed, cold climate may promote intrinsic virulence of these air borne pathogens, but may also modulate host antiviral immunity, causing a pivotal reduction of both adaptive and innate immune functions [6]. By considering this, early warming approaches could be beneficial in patients with moderate COVID-19, in order to prevent progression to severe disease. However, it has been noticed that also hot ambient temperatures may weaken antiviral T cell responses [6], and this could be reminiscent of what happens in virus-associated hyper-inflammatory syndromes, namely secondary hemophagocytic lymphohistiocytosis (sHLH) and macrophage activation syndrome (MAS), characterized by cytokine storms, unremitting high fever, and impaired virus control by specific T lymphocytes. In turn, following the “Goldilocks principle”—*neither too cold, neither too hot*—already well recognized in a variety of natural and biological phenomena, active temperature management may provide the right range of body temperature to maximize specific antimicrobial functions of the immune system, in particular, against pathogens inducing sepsis-like dysfunctional immunity.

## Data Availability

N/A
